# Effect of Vitamin D3 Supplementation on Respiratory Tract Infections in Healthy Individuals: A Systematic Review and Meta-Analysis of Randomized Controlled Trials

**DOI:** 10.1371/journal.pone.0162996

**Published:** 2016-09-15

**Authors:** Danielle Vuichard Gysin, Dyda Dao, Christian Michael Gysin, Lyubov Lytvyn, Mark Loeb

**Affiliations:** 1 Department of Clinical Epidemiology and Biostatistics, McMaster University, Hamilton, Ontario, Canada; 2 McMaster University, St. Joseph's Healthcare Hamilton, Hamilton, Ontario, Canada; 3 Department of Child Health and Evaluative Sciences, The Hospital for Sick Children, Toronto, Ontario, Canada; 4 Department of Pathology and Molecular Medicine, McMaster University, Hamilton, Ontario, Canada; 5 Department of Medicine, McMaster University, Hamilton, Ontario, Canada; 6 Institute for Infectious Diseases Research, McMaster University, Hamilton, Ontario, Canada; Hunter College, UNITED STATES

## Abstract

**Objective:**

Vitamin D supplementation may be a simple preventive measure against respiratory tract infections (RTIs) but evidence from randomized controlled trials is inconclusive. We aimed to systematically summarize results from interventions studying the protective effect of vitamin D supplementation on clinical and laboratory confirmed RTIs in healthy adults and children.

**Methods:**

Medline, EMBASE, CENTRAL, and CINAHL were screened from inception until present (last updated in January 2016) completed by a search of the grey literature, clinical trial registers and conference abstracts. We included randomized trials comparing vitamin D versus placebo or no treatment. Two independent reviewers were responsible for study selection and data extraction. Cochrane’s risk of bias tool and the GRADE approach were used for quality assessment. Estimates were pooled with random-effects models. Heterogeneity was explored by sub-group and meta-regression analyses.

**Results:**

Of 2627 original hits, 15 trials including 7053 individuals were ultimately eligible. All used oral cholecalciferol. We found a 6% risk reduction with vitamin D3 supplementation on clinical RTIs, but the result was not statistically significant (RR 0.94; 95% CI 0.88 to 1.00). Heterogeneity was large (I-square 57%) and overall study quality was low. There were too few studies to reliably assess a potential risk reduction of laboratory confirmed RTI. Evidence was insufficient to demonstrate an association between vitamin D supplementation and risk of clinical RTI in sub-groups with vitamin D deficiency.

**Conclusions:**

In previously healthy individuals vitamin D supplementation does not reduce the risk of clinical RTIs. However, this conclusion is based on a meta-analysis where the included studies differed with respect to population, baseline vitamin D levels and study length. This needs to be considered when interpreting the results. Future trials should focus on vitamin D deficient individuals and apply more objective and standardized outcome measurements.

## Introduction

Viral respiratory infections due to influenza and other respiratory viruses affect millions of people annually [1, 2]. The severity of illness can range from relatively mild, such as the common cold in healthy adults, to an increased risk of complications among small children and increased mortality in elderly people [3–5]. Winter epidemics of viral respiratory infections are associated with high rates of absenteeism and loss of work place productivity [6]. Although vaccines against influenza are available, their efficacy is reduced in mismatched seasons, and vaccines for other viral respiratory pathogens are not available [7, 8]. Behavioral measures, such as hand washing, may be used to reduce the burden, however their effectiveness is uncertain as is compliance [9, 10].

Expression of antimicrobial peptides, such as cathelicidin, which play an important role in the regulation of the innate immune system, is dependent on vitamin D [11]. It follows that vitamin D insufficiency can interfere with an adequate response to inflammation and infection. Moreover, vitamin D can inhibit inflammatory cytokines that are upregulated during influenza virus infection [12]. Vitamin D is therefore thought to have a role in both preventing and reducing the burden of viral respiratory infections.

Observational studies have shown an inverse correlation between 25-hydroxyvitamin D [25(OH)D] levels and the occurrence of respiratory tract infections in both children and adults [13–15]. The risk of confounding caused by inadequate adjustment is, however, a major limitation of observational study designs.

Five systematic reviews have summarized randomized controlled trials on the use of vitamin D to prevent respiratory infections [16–20]. Two of the five reviews did not perform a meta-analysis either due to a paucity of studies [20] or due to heterogeneity [18]. Charan and colleagues assessed five randomized controlled trials (RCTs) and found a protective effect of Vitamin D on RTIs in children and adults [OR 0.58, 95% confidence interval (CI) 0.42–0.81] [17]. A more recent review, that included 11 randomised placebo-controlled studies, reported a pooled OR of 0.64 (95% CI, 0.49–0.84) for a preventive effect of vitamin D on RTIs [16]. However, this review is limited by the fact that the outcome was a composite of direct and indirect measures of RTI. Both reviews showed marked clinical heterogeneity among the included trials.

Since publication of the last meta-analysis [16], 11 other randomized trials studying the preventive effect of vitamin D on RTIs have been reported. The disease burden, lack of consistent findings, and new available evidence justify another systematic review to assess potential benefit.

The goal of this systematic review was to summarize the RCT evidence comparing vitamin D supplementation to placebo or no treatment on the prevention of RTIs, the duration and severity of RTIs, absenteeism due to RTIs, and potential adverse effects in healthy populations.

## Methods

A protocol was established before start of the data extraction process and is available on Open Science Framework (https://osf.io/tyzvs/). The manuscript was prepared according to the statement of Preferred Reporting Items for Systematic Review and Meta-Analysis Protocols (PRISMA-P) 2015 [21] (S1 Methods).

### Literature search

Electronic databases were searched for trials that met our criteria without any restriction to language or year of publication: CENTRAL (The Cochrane Central Register of Controlled trials, from inception to February 12, 2015), MEDLINE (1966 to February 12, 2015) and EMBASE (1974 to February 12, 2015) through the OVID interface, and CINAHL through EBSCO (1981 to February 14, 2015), with weekly updates until January 4, 2016. Additionally, a search in the OpenGrey database (http://www.opengrey.eu/) was performed for any potential studies. Cited references from retrieved studies and reviews were crosschecked, and the International Clinical Trials Registry Platform (http://apps.who.int/trialsearch/) was scanned for ongoing trials. Principal investigators from completed but unpublished trials, as well as first authors from conference abstracts, were directly contacted for obtaining the full manuscript if available.

The following search terms using subject headings and variations on keywords were applied to all databases: vitamin D, cholecalciferol, ergocalciferol, calcifediol, hydroxyvitamin, dihydrotachysterol, alphacalcidol, respiratory tract infection, influenza, flu, common cold, and pneumonia (S2 Methods).

### Study selection

Randomized controlled trials were eligible with no restrictions on language or publication year. Healthy individuals of any age were eligible for inclusion given that various underlying disorders (e.g. osteoporosis, corticosteroid treatment in pulmonary obstructive disease, rheumatoid arthritis, or any other forms of compromised immune system) would either qualify those affected for vitamin D supplementation or render them more susceptible to RTIs.

The intervention was any form of vitamin D with either placebo or no treatment as comparator. Concomitant interventions were accepted as long as the type, dosage, and duration of application were identical in both the intervention and comparator arm.

The two primary outcomes of interest were: first episode of clinical RTI reported as cold or influenza-like illness with or without formal adjudication by medical personnel, and first episode of laboratory confirmed RTI assessed by standard microbiological methods. Secondary outcomes were the duration and severity of RTI-symptoms, and absenteeism due to RTI. Side effects of treatment included hypercalcemia, hyperphosphatemia, and the development of urinary tract stones.

Trials that met inclusion criteria were excluded if the intervention and/or comparator arm included a compound of micronutrients or multivitamin since it would be impossible to infer a single beneficiary effect to vitamin D. Studies with mycobacterial or fungal respiratory infections as the sole outcome were also excluded.

### Data abstraction and synthesis

Essential data were extracted, where possible, for each treatment arm individually. For studies with different time points of measurement data were extracted only at the time point closest to the end of the intervention. Because of the half-life of vitamin D [22], we did not record outcomes measured later than one month after the last administration in trials where participants received daily supplementations. For trials with bolus delivery, the latest time point considered acceptable for outcome measurement was at the end of the administration interval. The process of study selection and data abstraction was performed by two independent reviewers (D.V.G. and C.M.G. or D.V.G. and D.D.) using piloted screening forms. Disagreements were resolved by consensus. Trial authors were contacted for clarification or obtaining additional information.

Two independent reviewers (D.V. and D.D.) assessed the risk of bias using the Cochrane’s collaboration tool [23]. Sequence generation, concealment of allocation, and completeness of follow-up were evaluated at the study level. With respect to performance bias we referred to the primary outcome clinical RTI since the other outcomes were deemed at lower risk. Contents of trial protocols were compared with those in the publication to estimate the risk of reporting bias. If insufficient information was provided, the authors judged the specific risk of bias as unclear. A third reviewer (L.L.) was consulted to solve discrepancies.

### Statistical analyses

Study results were quantitatively combined for each outcome. For dichotomous outcomes we calculated pooled estimates expressed as relative risks (RR) with the corresponding 95% confidence intervals (CIs) using the Mantel-Haenszel method. The duration of symptoms and the number of days absent were summarized as mean differences (MDs) using the inverse variance method. Where necessary, we converted geometric means and CIs or medians and ranges into means and standard deviations (SDs) using published formulas [24, 25]. Due to the different types of reported estimates of symptom severity, we combined the corresponding log odds ratios and standard errors of the log odds ratio using the inverse variance method. We considered primarily data from intention to treat analyses or data with estimates from multiple imputation.

Two trials [26, 27] had more than one treatment group, but only one non-treatment group. Data from the treatment groups were therefore collapsed to compute the effect size to avoid double-counting of individuals or violation of independency. We quantified heterogeneity using the I^2^-statistic [28] and applied a random-effects model (DerSimonian and Laird) for all summary statistics [29].

We planned further exploration with sensitivity analyses excluding studies with high risk of bias for the primary outcome, and by deleting each study in turn to examine the magnitude of change in the summary effect. If results changed significantly, we pre-specified that corresponding studies had to be scrutinized further to explore heterogeneity. We planned a subgroup analysis to study whether the effect of vitamin D on clinical RTIs is different in participants with a low baseline 25(OH)D level (≤ 20ng/mL corresponding to ≤ 50 nmol/L) compared to participants with sufficient (> 20ng/mL) levels applying a test for interaction [30]. Another subgroup analysis evaluated whether the effect of vitamin D on clinical RTIs varied with different administration intervals (daily/weekly vs. monthly/three-monthly). We performed a meta-regression analysis of the log risk ratio of clinical RTI on the average daily dosage of vitamin D.

### Evaluation of the overall body of evidence

Risk of publication bias was visually and formally assessed with a funnel plot and Egger’s test [31], respectively. Data were analyzed using the statistical software packages Review Manager (version 5.3) and Comprehensive Meta-Analysis (version 3.0). We assessed the overall confidence in the estimate applying the criteria published by the GRADE working group [32]: risk of bias, inconsistency, indirectness, imprecision, and publication bias. We integrated the evidence profile in the summary of findings table using GRADE profiler software.

## Results

**Fig 1:**
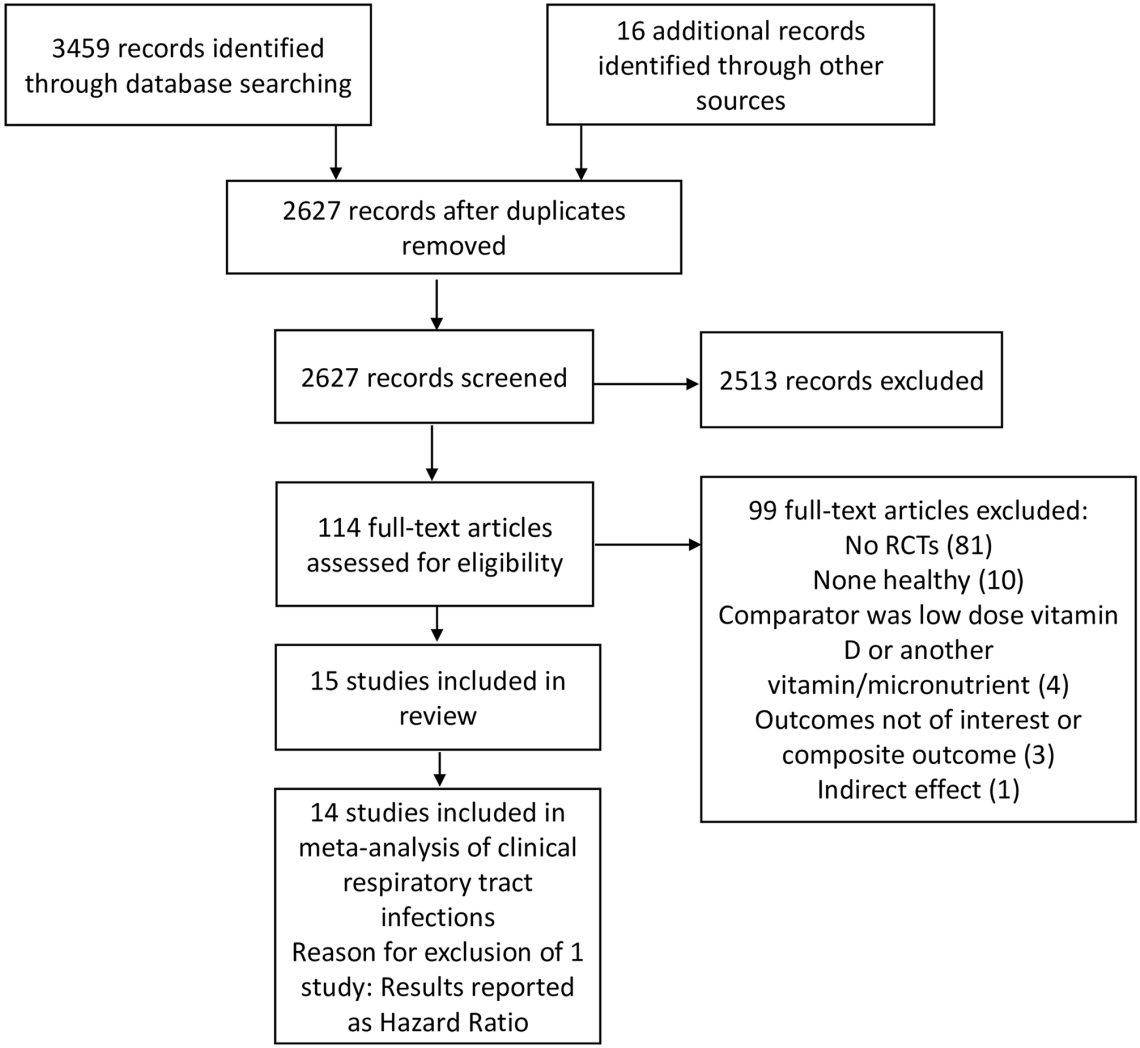
PRISMA flow diagram of the study selection process.

Of the initial 2627 potentially relevant citations, 114 full texts were screened and 15 studies were deemed eligible (Fig 1 and S1 Table). A total of 7053 healthy individuals were randomized, with 50.6% women, and the median age was 19 years (IQR 10–49). Ten studies investigated adolescents or adults [33–42]; two included only infants [26, 43], two were conducted in school-aged children [44, 45], and one assessed solely elderly people [27]; the latter was presented as abstract. Complete data were available for 7001 cases. Baseline 25(OH)D serum levels were measured in 11 studies, and in four of these trials (73% of the participants) that measured 25(OH)D serum levels average baseline levels were insufficient (≤ 20 ng/mL) [27, 33, 43, 44]. All trials administered cholecalciferol (vitamin D3) orally. Average daily dose was 1500 IU (range: 300 to 3700 IU). Frequency of administration ranged from daily to every three months. All but one trial [34] used a placebo as comparator. Trial duration varied between seven weeks and three years (median 17 weeks) (Table 1).

**Table 1:** Characteristics of included studies.

Author, year [Ref.]	Study design, Duration*, Country	No. rando-mized	Age, mean (SD)	Women (%)	Baseline mean 25(OH)D levels (in ng/mL)	Oral vitamin D3 (cholecalciferol) (in mcg)	Control	Interval	Outcomes included in review
**Aloia, 2007** [33]	Post-hoc analysis of RCT, 3 years, U.S.	E: 104	E: 59.9 (6.2)	E: 100	E: 19.3 (8.4)	Years 1–2: 20 mcg Year 3: 50 mcg Plus calcium 1200–1500 mg	Placebo plus calcium 1200–1500 mg	daily	Clinical RTI
		C: 104	C: 61.2 (6.3)	C: 100	C: 17.2 (6.6)				
**Camargo, 2012** [44]	Sub-study of a cluster RCT, 7 weeks, Mongolia	E: 141	E: 10.1 (0.9)	E: 46	E: 7.0 (5.0–9.9)	Mongolian milk with 7.5 mcg	Non fortified milk	daily	Clinical RTI
		C: 103	C: 9.8 (1.0)	C: 50	C: 6.8 (4.3–9.5)‡				
**De Gruijl, 2012** [34]	RCT, 8 weeks, The Netherlands	E: 37	E: 21.9 (2.3)	E: 89	E: 23.2 (7.2)	25 mcg	no treatment	daily	Clinical RTI
		C: 33	C: 21.5 (2.1)	C: 94	C: 24.8 (9.6)				
**Dubnov-Raz, 2014** [35]	RCT, 12 weeks, Israel	E: 28	E: 15.2 (1.3)	E: 39	E: 24.3 (4.9	50 mcg	Placebo	daily	Clinical RTI, mean duration & severity
		C: 27	C: 15.1 (1.9)	C: 33	C: 24.4 (4.7)				
**Goodall, 2014** [36]	RCT, 2x2 factorial design, 8 weeks, Canada	E: 300	E: 19 (18–21)	E: 64	n.d.	250 mcg +/- gargling	Placebo +/- gargling	weekly	Clinical & lab. confirmed RTI, mean duration & severity
		C: 300	C: 19 (18–21)	C: 63					
**Grant, 2015** [26]	Ad hoc analysis of a RCT, 12 months#, New Zealand	E1: 87	E1: 0	E1: 51	E1: 24 (23–24.5)	E1: 10 mcg	Placebo	daily	Clinical RTI
		E2: 86	E2: 0	E2: 56	E2: 27.5 (26–28)	E2: 20 mcg**			
		C: 87	C: 0	C: 48	C: 15 (12–17)‡				
**Laaksi, 2010** [37]	RCT, 6 months, Finland	E: 80	18–28 years	E: 0	E: 31.5 (6.0)	10 mcg	Placebo	daily	Clinical RTI, no. days absent
		C: 84		C: 0	C: 29.8 (8.3)‡‡				
**Li-Ng, 2009** [38]	RCT, 3 months, U.S.	E: 84	E: 59.3 (13)	E: 78	E: 25.7 (10.2)	50 mcg	Placebo	daily	Clinical RTI, mean duration & severity
		C: 78	C: 58.1 (13.4)	C: 81	C: 25.2 (10.3)				
**Manasek, 2012** [43]	RCT, 18 months, Afghanistan	E: 1524	E: 0.5	E: 47	E: n.d.	2500 mcg	Placebo	every 3 months	Clinical RTI
		C: 1522	C: 0.5	C: 49	C: 16 (11–21)##				
**Murdoch, 2012** [39]	RCT, 18 months, New Zealand	E: 161	E: 47 (10)	E: 75	E: 29 (9)	At 0 and 1 month: 5000 mcg, then 2500 mcg	Placebo	Every month	Clinical RTI, lab. confirmed mean duration & severity, no. days absent
		C: 161	C: 48 (10)	C: 75	C: 28 (9)				
**Neale, 2013** [27]	Cross-sectional study nested within RCT, 12 months, Australia	E1: 215	60–84 years	E1: 46	E1: 16.6 (5.1)	E1: 750 mcg	Placebo	every month	Clinical RTI
		E2: 215		E2: 47	E2: 16.6 (5.6)	E2: 1500 mcg			
		C: 214		C: 46	C: 16.8 (5.3)				
**Rees, 2013** [40]	RCT sub-study, 2x2 factorial design, 17 months, USA	E: 399	E: 60.7 (6.7)	E: 41	E: 33.3 (9.9)	25 mcg +/- calcium 1200mg	Placebo +/- calcium 1200mg	daily	Clinical RTI, mean duration
		C: 360	C: 60.5 (6.4)	C: 44	C: 25.1 (9.1)				
**Simpson, 2015** [41]	RCT, 17 weeks, Australia	E: 18	E: 30.3 (11.8)	E: 50	E: 24.2 (5.6)	500 mcg	Placebo	weekly	Duration, severity
		C: 16	C: 35 (12.5)	C: 69	C: 30.6 (10.9)				
**Urashima, 2010** [45]	RCT, 4 months, Japan	E: 217	E: 10.0 (2.2)	E: 43	n.d.	30 mcg	Placebo	daily	Clinical RTI, lab. confirmed
		C: 213	C: 10.4 (2.4)	C: 45					
**Urashima, 2014** [42]	RCT, 8 weeks, Japan	E: 148	15–18 years	E: 33	n.d.	50 mcg	Placebo	daily	Clinical RTI, lab. confirmed, no. days absent
		C: 99		C: 35					

* If not otherwise indicated, study duration is identical with duration of intervention

^‡^ Medians with interquartile range (IQR)

^#^ 12 months = follow-up of infants, Total duration of intervention: 27 weeks gestation + until infants were 6 months

**Infant dosages only are indicated

^‡‡^ Measured in 29 (experimental group) and 44 (control group) men, respectively

^##^ Cord blood measurements

Abbreviations: C: control group; E: experimental group(s); IU: international units; n.d.: no data; RCT: randomized controlled trial; RTI: respiratory tract infection

Units of measurement, if not otherwise indicated: Age is reported in years, Vitamin D serum levels are indicated as ng/ml (where necessary, conversion from nmol/L to ng/ml was performed using factor: 0.4), Vitamin D dose is indicated in microgram (mcg) where 0.025 mcg cholecalciferol is equivalent to 1 IU cholecalciferol.

**Fig 2:**
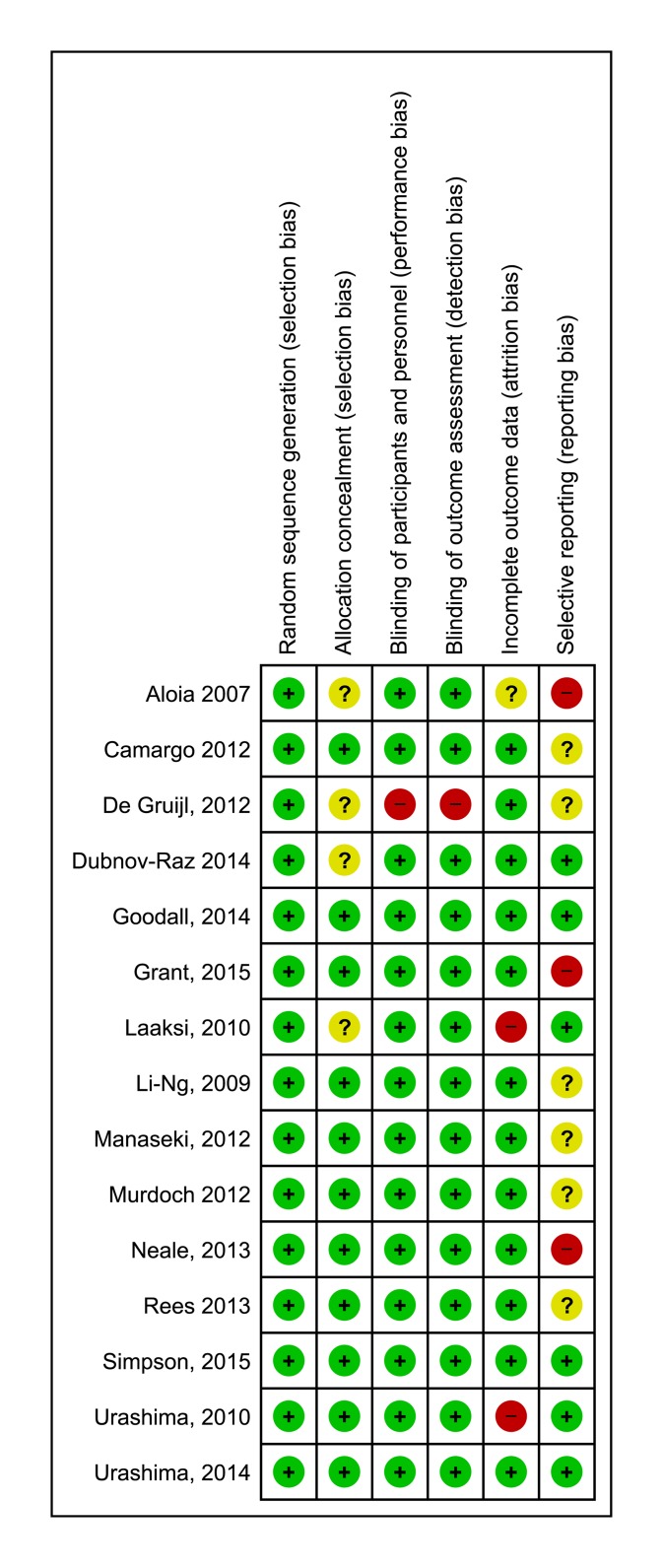
Risk of bias summary: review authors' judgements about each risk of bias item for each included study.

Risk of bias in individual studies was moderate to low: three trials [26, 27, 33] were deemed at high risk for selective reporting, one study [34] was judged at high risk for performance bias due to its open-labelled design (Fig 2).

### Risk of clinical and laboratory confirmed RTI

**Fig 3:**
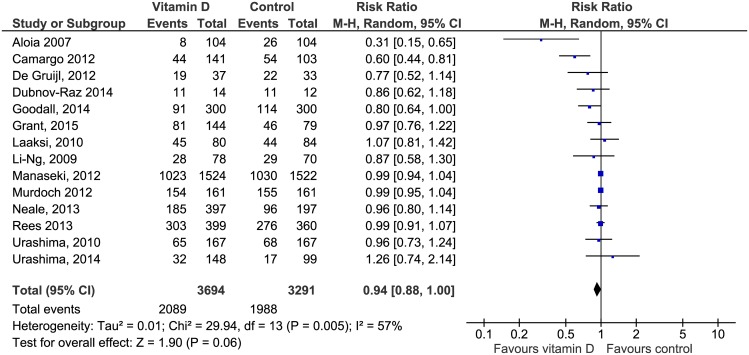
Forest plot of comparisons of vitamin D versus control on clinical RTI. M-H = Mantel-Haenzsel statistics, Random = random effects model.

**Fig 4:**
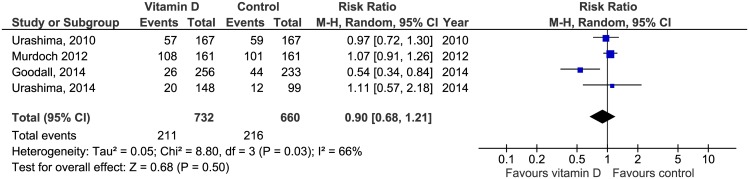
Forest plot of comparisons of vitamin D versus control on laboratory confirmed RTI. M-H = Mantel-Haenzsel statistics, Random = random effects model.

There were a total of 4077 first episodes of clinical RTIs in 14 studies. Baseline risk in the control group was 60.4%. The pooled risk of experiencing an RTI was 6% lower in the vitamin D group compared to the non-treatment group, but the result was not statistically significant (RR 0.94; 95% CI 0.88 to 1.00) (Fig 3). Between study variability was large (I2 = 57%). We found a 10% lower risk of a first laboratory-confirmed viral respiratory infection in the vitamin D group versus the comparator group. The result was not statistically significant (RR 0.90; 95% CI 0.68 to 1.21) and heterogeneity was large (I2 = 66%) (Fig 4). Of the four studies assessing this outcome, two tested only for influenza virus A and B [42, 45] whereas the other two studies [36, 39] applied a diagnostic panel comprising 16 respiratory viruses and encountered predominantly rhinovirus cases.

### Symptom duration, absenteeism and severity of RTI

**Fig 5:**
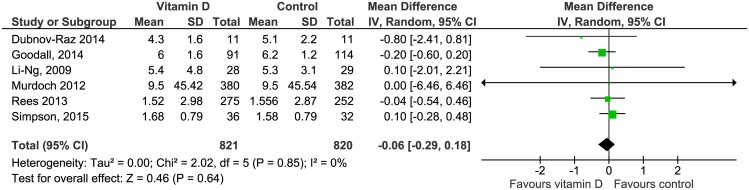
Forest plot of comparison vitamin D versus control on mean duration of RTI symptoms. IV = inverse variance method, Random = random effects model.

The pooled summary estimate of six studies [35, 36, 38–41] showed a marginal mean reduction in symptom duration (MD -0.06 days; 95% CI -0.29 to 0.18) in the vitamin D group compared to placebo, which was not statistically significant (Fig 5).

**Fig 6:**
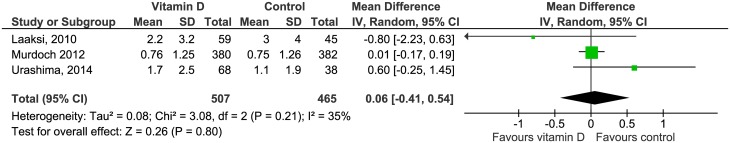
Forest plot of comparisons vitamin D versus control on number of days absent from work/school (absenteeism) due to RTI. IV = inverse variance method, Random = random effects model.

The three studies [37, 39, 42] which measured the number of sick days leading to absence from duty or school yielded highly inconsistent results: The pooled mean difference was 0.06 days in favour of placebo but with a 95% CI crossing the null (95% CI -1.33 to 1.37) (Fig 6).

**Fig 7:**
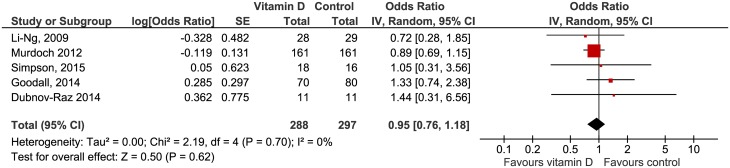
Forest plot of comparisons vitamin D versus control on severity of RTIs. IV = inverse variance method, Random = random effects model.

Five studies [35, 36, 38, 39, 41] assessed severity of RTI symptoms on various scales. Definitions applied to assess symptom severity were as follows: the average self-rated score of symptom severity on a scale of 1 to 7 (1 = very mild, 3 = mild, 5 = moderate, 7 = severe) [35], the sum of seven consecutive daily severity scores [36, 39], and the mean severity score on a scale from 1–5 (1 = healthy and 5 = very ill) [38] and 0–5 (0 = no symptoms and 5 = most severe symptoms) [41], respectively. The pooled OR of 0.95 (95% CI 0.76 to 1.18) suggests less pronounced severity in the vitamin D group, but this result did not reach statistical significance (Fig 7).

### Adverse events

Six studies specifically addressed at least one of the following: hypercalcemia [27, 33], hyperphosphatemia [41, 42, 45], or nephrolithiasis [33, 38, 41, 42, 45]. Nine of ten cases with mild hypercalcemia occurred in the same trial with concomitant calcium administration [33]. There were no identified cases of either hyperphosphatemia or nephrolithiasis.

### Additional analyses

Although the RR of 0.77 (95% CI 0.59–1.02) suggests a probable beneficiary effect of vitamin D on clinical RTI among those with low (≤ 20 ng/ml) versus sufficient (> 20 ng/ml) baseline 25(OH)D levels, the test for subgroup differences, however, was not statistically significant (P = .08). There was a lower risk of clinical RTIs among those with daily/weekly vitamin D supplementation (RR 0.87; 95% CI 0.76 to 0.99) versus those with less frequent (monthly or three-monthly) bolus administration (RR 0.99; 95% CI 0.96 to 1.02), but the difference was not statistically significant (P = .06). Average daily dosage turned out to be a non-significant predictor in the univariate model evaluating the effect of vitamin D on clinical RTI (P = .49) and thus, could not explain the between-study variance (R-squared analog = 0.00) (S1–S3 Figs).

### Risk of bias across studies

We observed large heterogeneity for the two primary outcomes. We could not identify one particular study of major influence by removing one study at a time. Both, the aspect of the funnel plot and Egger’s test (P = .03) suggested possible publication bias (S4 and S5 Figs). Due to the low number of studies in the secondary outcomes we suspected ubiquitous risk of publication bias and therefore downgraded the overall quality of evidence for each individual outcome (S2 Table).

## Discussion

In this meta-analysis we were unable to demonstrate a significant beneficiary effect of vitamin D versus placebo or no treatment on clinical RTI in a generally healthy population (RR 0.94, 95% CI 0.88 to 1.00). Vitamin D supplementation may potentially lower the risk of laboratory confirmed RTI and marginally reduce mean duration and severity of symptoms. However, neither of these pooled estimates were statistically significant. The confidence in the estimate of effect based on the overall body of evidence was considered to be low, mainly due to unexplained heterogeneity (inconsistency) in the primary outcomes and the probability of publication bias.

### How our results compare to previous reviews

Contrary to two previous meta-analyses [16, 17] in which vitamin D supplementation was associated with significantly less RTI events, our findings suggest no preventive effect of vitamin D, thereby confirming the pooled results of Mao and colleagues [19], which found no difference of vitamin D versus placebo in reducing the risk of RTIs in healthy individuals (RR 0.98, 95% CI 0.93–1.03).

Apart from including recently published evidence, our work differs from previous reviews by study selection and types of outcome. The exclusion of non-healthy people in our review, especially those with underlying pulmonary disorders and immune defects, could have mitigated against a more beneficiary effect of vitamin D as has been reported in the reviews by Charan et al. (OR 0.58; 95% CI 0.42–0.81) [17] and Bergman et al. (OR 0.64; 95% CI 0.49–0.84) [16]. Furthermore, rather than providing a summary of various measurements, our pooled effects were separately analyzed for a subjective endpoint (clinical RTIs) and several more objectively measurable outcomes (e.g. laboratory confirmed RTI). Allowance for low dose vitamin D supplementation in the meta-analysis by Mao et al. [19] potentially diluted the effect. Despite our more rigorous criteria to only include placebo or no treatment as comparator, we reached the same result. There are no previous meta-analyses pooling the effect of vitamin D on RTI-related absenteeism or the duration and severity of RTIs.

### Why vitamin D supplementation may not always work

Fabri et al. have shown in vitro that IFN-γ mediated expression of antimicrobial peptides (AMP) is dependent on the vitamin D concentration and that this pathway is inhibited in serum of vitamin D deficient Afro-Americans [46]. Our subgroup analysis and the previous work from others [18, 47, 48] suggest that a reduction of the risk of RTIs may indeed occur solely in the vitamin D insufficient/deficient state. Although, the largest trial in vitamin-D deficient infants did not show a difference in the number of first episodes of pneumonia [43], it can be argued that the bolus administration did not achieve constantly higher vitamin D serum concentrations which could be one clue for the ineffectiveness of this regimen. Indeed, our post hoc sub-group analysis and also an earlier analysis by Bergman and colleagues [16] suggest that less frequent bolus regimens are not effective in reducing the risk of RTI. Their rationale was that large bolus doses of 2500 mcg vitamin D (100’000 IU cholecalciferol equivalent) or more given at 1–3 months intervals might have an immunosuppressive rather than immune-stimulating effect. The recent findings from an RCT where intermittent high bolus dose vitamin D was less effective than daily low dose vitamin D supplementation in preventing RTI and even increased the risk of upper RTI seem affirmative [49]. It has been previously suggested that genetic factors may play a role [47]; however, vitamin D receptor polymorphisms as potential key players in the innate and acquired immune defense of respiratory tract infections [50] have not been investigated in these trials.

Lastly, in a healthy vitamin D replete population a clinically important effect from supplementation is unlikely.

### Strengths and limitations of the study

To our knowledge this review incorporates the largest body of evidence, comprising 15 randomized controlled trials and over 7000 healthy individuals. The search strategy was rigorous and clear eligibility criteria were applied. Another strength of this review is that different outcomes, rather than a composite outcome of various indirect and direct measures, were evaluated. All trials administered vitamin D3 (cholecalciferol) orally and adherence in most trials was reported to be high with a generally low loss to follow-up rate. Remaining heterogeneity was broadly explored by various sensitivity analyses.

We acknowledge that despite our efforts to confine our meta-analysis to healthy individuals there were still major differences across studies with respect to population, settings, dosing regimens and outcome measurements. Furthermore, our sub-group analyses should be interpreted with caution since they were done on a subset of studies or were performed in retrospect.

## Conclusions

We found no significant protective effect of vitamin D supplementation on clinical RTI in otherwise healthy people of various ages. The overall level of evidence was, however, low. Evidence seems insufficient to determine the comparative effectiveness of vitamin D versus placebo with regard to laboratory confirmed RTI. In view of the burden common colds and influenza like illness cause more rigorous research is needed.

Large randomized and placebo controlled trials in selected patient groups investigating the effectiveness of vitamin D supplementation on laboratory confirmed RTI are needed. If diagnostic testing is not available, using a more uniform and standardized definition of clinical RTI based on a validated assessment would be desirable. Measurement of 25(OH)D levels at baseline and follow-up should be standard. Elderly people and (pre-) school-aged children, as well as obese patients were underrepresented in this systematic review and deserve more attention in future trials. Our systematic review showed that in healthy populations, vitamin D supplementation is unlikely to show any benefit. Although the findings are negative, we believe that may be of value to both clinicians and policy makers so that they fully understand the lack of evidence to support additional vitamin D supplementation in healthy populations with adequate levels of vitamin D. Health care providers should continue to follow guidelines on 25(OH)D-level guided vitamin D supplementation for maintenance of bone and general health [51, 52]. This meta-analysis does not support any specific changes in guidelines.

## Supporting Information

S1 Fig Forest plot of comparison vitamin D versus control in subgroups with insufficient (≤ 20 ng/ml) vs. sufficient (> 20 ng/ml) 25(OH)D levels on clinical RTI. 25-OH = 25-hydroxy, M-H = Mantel-Haenzsel statistics, Random = random effects model.
(DOCX)

S2 Fig Forest plot of comparison: Vitamin D versus control, outcome: Clinical RTI. Subgroup analysis according to application interval. M-H = Mantel-Haenzsel statistics, Random = random effects model.
(DOCX)

S3 Fig Univariate random-effects meta-regression of clinical RTI (log risk ratio) on average daily dosage. (DOCX)

S4 Fig Sensitivity analysis for the effect of vitamin D on clinical RTI removing one study in turn. (DOCX)

S5 Fig Funnel plot of comparison vitamin D versus control on clinical RTI (random effects model). (DOCX)

S1 Methods PRISMA checklist. (DOC)

S2 Methods Detailed search strategy. (DOCX)

S1 Table List of excluded RCTs. (DOCX)

S2 Table GRADE level of evidence and summary of findings of Vitamin D for the prevention of RTIs.table. (DOCX)
