# Supplementary material for: Effect of Vitamin D3 Supplementation on Respiratory Tract Infections in Healthy Individuals: A Systematic Review and Meta-Analysis of Randomized Controlled Trials
Source: PLoS One. 2016 Sep 15;11(9):e0162996. doi: 10.1371/journal.pone.0162996 (PMC5025082; doi:10.1371/journal.pone.0162996)
Supplement: S1 Fig — 25-OH = 25-hydroxy, M-H = Mantel-Haenzsel statistics, Random = random effects model. (DOCX) [file pone.0162996.s001.docx]

**Systematic review and meta-analysis on the effect of vitamin D supplementation on respiratory tract infection in healthy individuals.**

Danielle Vuichard Gysin, Dyda Dao, Christian Michael Gysin, Lyubov Lytvyn, Mark Loeb


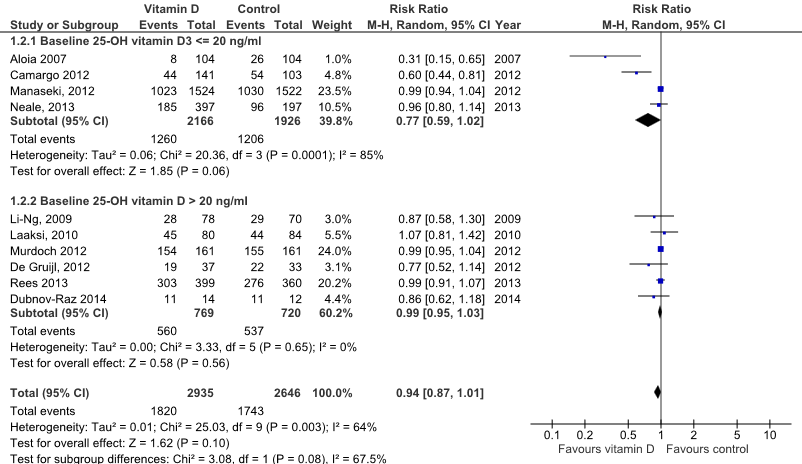
**S1 Figure. Forest plot of comparison vitamin D versus control in subgroups with insufficient (≤ 20 ng/ml) vs. sufficient (> 20 ng/ml) 25(OH)D levels on clinical RTI**

M-H = Mantel-Haenzsel statistics, Random = random effects model.
