# Supplementary material for: Effect of Vitamin D3 Supplementation on Respiratory Tract Infections in Healthy Individuals: A Systematic Review and Meta-Analysis of Randomized Controlled Trials
Source: PLoS One. 2016 Sep 15;11(9):e0162996. doi: 10.1371/journal.pone.0162996 (PMC5025082; doi:10.1371/journal.pone.0162996)

**Systematic review and meta-analysis on the effect of vitamin D supplementation on respiratory tract infection in healthy individuals.**

Danielle Vuichard Gysin, Dyda Dao, Christian Michael Gysin, Lyubov Lytvyn, Mark Loeb

**S3 Fig. Univariate random-effects meta-regression of clinical RTI (log risk ratio) on average daily dosage.**


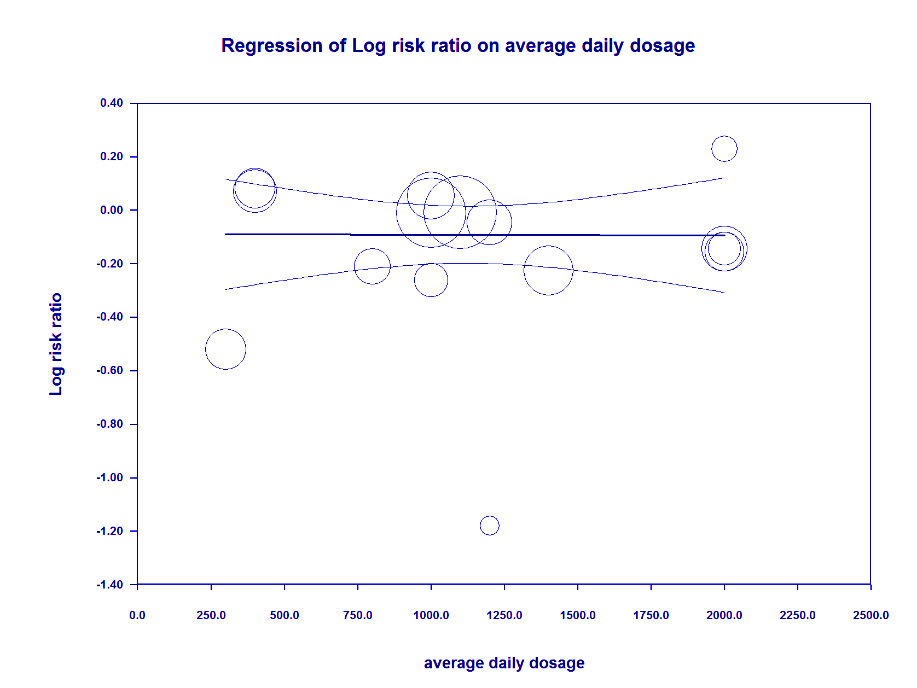

Supplement: S3 Fig — (DOCX) [file pone.0162996.s003.docx]
