# Supplementary material for: Effect of Vitamin D3 Supplementation on Respiratory Tract Infections in Healthy Individuals: A Systematic Review and Meta-Analysis of Randomized Controlled Trials
Source: PLoS One. 2016 Sep 15;11(9):e0162996. doi: 10.1371/journal.pone.0162996 (PMC5025082; doi:10.1371/journal.pone.0162996)
Supplement: S2 Methods — (DOCX) [file pone.0162996.s007.docx]

**Systematic review and meta-analysis on the effect of vitamin D supplementation on respiratory tract infection in healthy individuals.**

Danielle Vuichard Gysin, Dyda Dao, Christian Michael Gysin, Lyubov Lytvyn, Mark Loeb

**S2 Methods. Detailed search strategy.**

MEDLINE search through OVID-interface (1946 to 12^th^ Feb 2015 with weekly updates until 4^th^ Jan 2016):

1. exp Vitamin D/
2. Vitamin D?2.mp.
3. Vitamin D?3.mp.
4. vitamin* D?.mp.
5. cholecalciferol*.mp.
6. colecalciferol*.mp.
7. ergocalciferol*.mp.
8. calciferol*.mp.
9. calcifediol*.mp.
10. hydroxyvitamin*.mp.
11. 25 hydroxyvitamin D3.mp.
12. dihydrotachysterol*.mp.
13. alphacalcidol*.mp.
14. alfacalcidol*.mp.
15. 1-14/OR
16. exp Respiratory Tract Infections/
17. exp Influenza, Human/
18. exp Common Cold/
19. exp Pneumonia/
20. flu.mp.
21. flu like syndrom*.mp.
22. flu like symptom*.mp.
23. Respiratory tract infection*.mp.
24. respiratory.mp.
25. respiratory infection*.mp.
26. common cold.mp.
27. influenza.mp.
28. influenza like illness*.mp.
29. rhinit*.mp.
30. bronchit*.mp.
31. bronchopneumonia.mp.
32. 16-31/OR
33. 15 AND 32

**S1 Table. List of excluded RCTs.**

| **Study** | **Reason for exclusion** |
| --- | --- |
| **Avenell, 2007^1^** | Outcomes of interest were not specifically addressed. |
| **Bartram, 2003^2^** | Non-healthy population, other micronutrients and none of the outcomes of interest assessed. |
| **Behnamfar, 2011^3^** | Comparator was vitamin E |
| **Bergmann, 2012^4^** | Of the included participants, there were 80% with either a documented immunoglobulin deficiency or CVID and approximately half of the participants suffered from an underlying pulmonary disease including asthma, bronchiectasis or COPD |
| **Choudhary, 2012^5^** | Population had underlying severe pneumonia. |
| **Fort, 2015^6^** | Extremely preterm infants were deemed as non-healthy population. |
| **Gianni, 2014^7^** | Non-healthy population (preterm infants) and low dose vitamin D in control group. |
| **Goldring, 2013^8^** | Studied indirect effect: the pregnant mother and not the baby received the intervention |
| **Jain, 2002^9^** | Comparator was other vitamins or micronutrients |
| **Jorde, 2012^10^** | Non-healthy population. Authors state in their discussion section: "only a few of our subjects were completely healthy", ..."results may not apply for a more healthy population." |
| **Majak, 2011^11^** | Non-healthy population. |
| **Manaseki, 2010^12^** | Studied children admitted to hospital with pneumonia. |
| **Martineau, 2013^13^** | Low-dose vitamin D as comparator. |
| **McDonald, 2006^14^** | Non-healthy population. |
| **No author, trial no. NCT01103934^15^** | Study results available (unpublished) but none of the outcomes of interest investigated. |
| **Sazawal, 2007^16^** | other vitamins or micronutrients as comparator |
| **Sneve, 2008^17^** | None of the outcomes of interest addressed. |
| **Wu, 2012^18^** | Non-healthy population. |


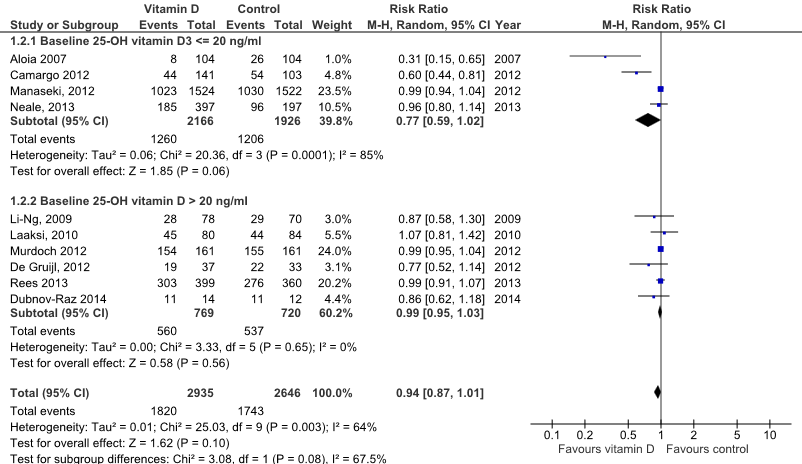
**S1 Figure. Forest plot of comparison vitamin D versus control in subgroups with insufficient (≤ 20 ng/ml) vs. sufficient (> 20 ng/ml) 25(OH)D levels on clinical RTI**

M-H = Mantel-Haenzsel statistics, Random = random effects model.

**S2 Figure. Forest plot of comparison: Vitamin D versus control, outcome: Clinical RTI. Subgroup analysis according to application interval.**


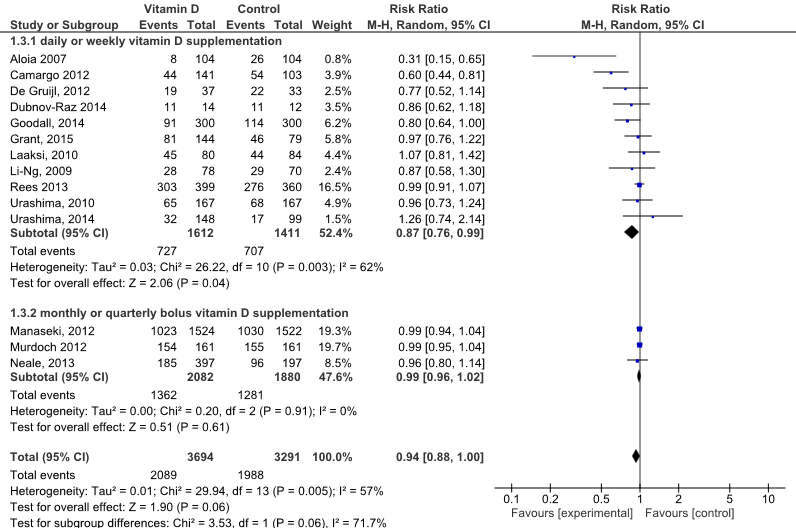


M-H = Mantel-Haenzsel statistics, Random = random effects model.

**S3 Figure. Univariate random-effects meta-regression of clinical RTI (log risk ratio) on average daily dosage.**


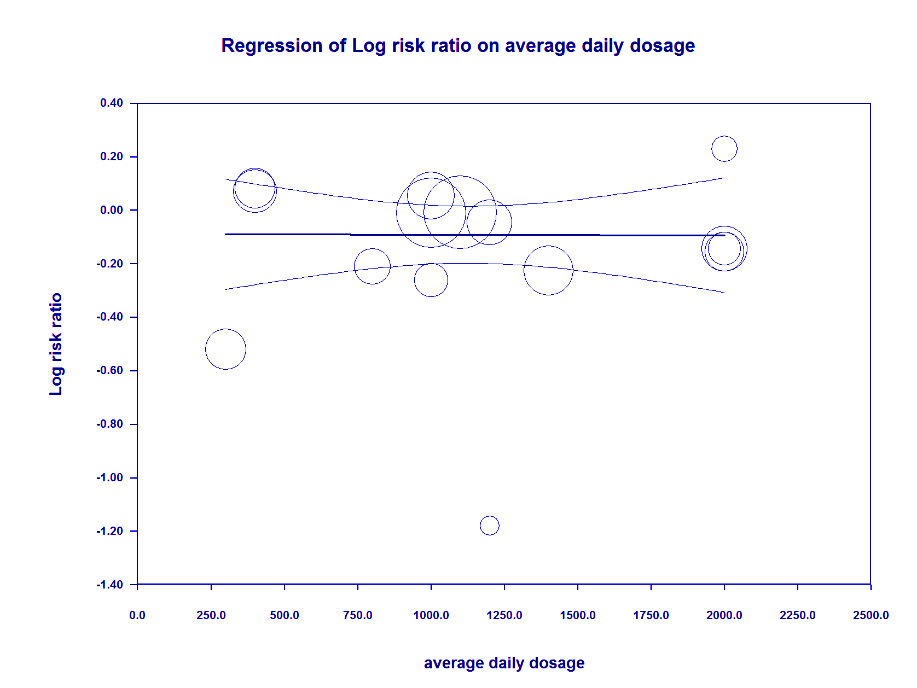


**S4 Figure. Sensitivity analysis for the effect of vitamin D on clinical RTI removing one study in turn.**

**S5 Fig. Funnel plot of comparison vitamin D versus control on clinical RTI (random effects model).**

**S2 Table. GRADE level of evidence and summary of findings of Vitamin D for the prevention of RTIs**

| **Quality assessment** | | | | | | | **Summary of Findings** | | | | |
| --- | --- | --- | --- | --- | --- | --- | --- | --- | --- | --- | --- |
| **Participants (studies) Follow up** | **Risk of bias** | **Inconsistency** | **Indirectness** | **Imprecision** | **Publication bias** | **Overall quality of evidence** | **Study event rates (%)** | | **Relative effect** (95% CI) | **Anticipated absolute effects** | |
|  |  |  |  |  |  |  | **With Control** | **With Vitamin D versus Control** |  | **Risk with Control** | **Risk difference with Vitamin D versus Control** (95% CI) |
| **Clinical Respiratory Tract Infection (RTI) (at least one episode):** | | | | | | | | | | | |
| 6985 (14 studies) 17 weeks | no serious risk of bias | serious^1^ | no serious indirectness | no serious imprecision | strongly suspected^2^ | ⊕⊕⊝⊝ **LOW**^1,2^ due to inconsistency, publication bias | 1988/3291  (60.4%) | 2089/3694  (56.6%) | **RR 0.94**  (0.88 to 1) | **Study population** | |
|  |  |  |  |  |  |  |  |  |  | **604 per 1000** | **36 fewer per 1000** (from 72 fewer to 0 more) |
|  |  |  |  |  |  |  |  |  |  | **Moderate** | |
|  |  |  |  |  |  |  |  |  |  | **524 per 1000** | **31 fewer per 1000** (from 63 fewer to 0 more) |
| **Laboratory confirmed RTI (at least one episode):** | | | | | | | | | | | |
| 1392 (4 studies) 12.5 weeks | no serious risk of bias | serious^1^ | no serious indirectness | no serious imprecision | strongly suspected^3^ | ⊕⊕⊝⊝ **LOW**^1,3^ due to inconsistency, publication bias | 216/660  (32.7%) | 211/732  (28.8%) | **RR 0.9**  (0.68 to 1.21) | **Study population** | |
|  |  |  |  |  |  |  |  |  |  | **327 per 1000** | **33 fewer per 1000** (from 105 fewer to 69 more) |
|  |  |  |  |  |  |  |  |  |  | **Moderate** | |
|  |  |  |  |  |  |  |  |  |  | **271 per 1000** | **27 fewer per 1000** (from 87 fewer to 57 more) |
| **Mean duration (in days) of RTI (any episode):** (Better indicated by lower values) | | | | | | | | | | | |
| 1641 (6 studies) 14.5 weeks | no serious risk of bias | no serious inconsistency | no serious indirectness | serious^4^ | strongly suspected^3^ | ⊕⊕⊝⊝ **LOW**^3,4^ due to imprecision, publication bias | 820 | 821 | **-** | The mean duration of RTI in the intervention groups was **0.06 lower** (0.29 lower to 0.18 higher) | |
| **Mean number of sick days** (Better indicated by lower values) | | | | | | | | | | | |
| 972 (3 studies) 26 weeks | no serious risk of bias | serious^1^ | no serious indirectness | serious^4^ | strongly suspected^3^ | ⊕⊝⊝⊝ **VERY LOW**^1,3,4^ due to inconsistency, imprecision, publication bias | 465 | 507 | **-** |  | The mean number of sick days in the intervention groups was **0.06 higher** (0.41 lower to 0.54 higher) |
| **Severity of RTI** | | | | | | | | | | | |
| 585 (5 studies) 12 weeks | no serious risk of bias | no serious inconsistency | serious^5^ | no serious imprecision | reporting bias strongly suspected^3^ | ⊕⊕⊝⊝ **LOW**^3,4^ due to indirectness, publication bias | 0/297  (0%) | 0/288  (0%) | **OR 0.95**  (0.76 to 1.18) | **Study population** | |
|  |  |  |  |  |  |  |  |  |  | - | - |

^1^ Unexplained heterogeneity
^2^ Visual inspection of Funnel plot and Egger's test strongly suggest publication bias.
^3^ Low number of published studies did not allow formal assessment of publication bias.
^4^ Large confidence intervals.
^5^ Various definitions were applied to measure this outcome.

**References of excluded randomized controlled trials:**

1. Avenell A, Cook JA, Maclennan GS, Macpherson GC. Vitamin D supplementation to prevent infections: a sub-study of a randomised placebo-controlled trial in older people (RECORD trial, ISRCTN 51647438). *Age and ageing.* 2007;36(5):574-577.

2. Bartram SA, Peaston RT, Rawlings DJ, Francis RM, Thompson NP. A randomized controlled trial of calcium with vitamin D, alone or in combination with intravenous pamidronate, for the treatment of low bone mineral density associated with Crohn's disease. *Alimentary Pharmacology and Therapeutics.* 2003;18(11-12):1121-1127.

3. Behnamfar Z, Mehrdad S, Zahra B. Effect of maintenance dose (30000 unit per month) 25- Hydroxyvitamin D on Upper Respiratory Tract Infection in children of day care center. *European Journal of Medical Research.* 2011;16:49.

4. Bergman P, Norlin AC, Hansen S, et al. Vitamin D3 supplementation in patients with frequent respiratory tract infections: A randomised and double-blind intervention study. *BMJ open.* 2012;2(6).

5. Choudhary N, Gupta P. Vitamin D supplementation for severe pneumonia--a randomized controlled trial. *Indian pediatrics.* 2012;49(6):449-454.

6. Fort P, Salas AA, Ambalavanan N. Randomized clinical trial of vitamin D supplementation in extremely preterm infants. *Journal of Investigative Medicine.* 2015;63 (2):417.

7. Gianni ML, Roggero P, Amato O, et al. Randomized outcome trial of nutrient-enriched formula and neurodevelopment outcome in preterm infants. *BMC Pediatrics.* 2014;14(1).

8. Goldring ST, Griffiths CJ, Martineau AR, Robinson S, Yu C, Poulton S. Prenatal Vitamin D Supplementation and Child Respiratory Health: A Randomised Controlled Trial. *PloS one.* 2013;8(6):e66627.

9. Jain AL. Influence of vitamins and trace-elements on the incidence of respiratory infection in the elderly. *Nutrition Research.* 2002;22(1-2):85-87.

10. Jorde R, Witham M, Janssens W, et al. Vitamin D supplementation did not prevent influenza-like illness as diagnosed retrospectively by questionnaires in subjects participating in randomized clinical trials. *Scandinavian journal of infectious diseases.* 2012;44(2):126-132.

11. Majak P, Olszowiec-Chlebna M, Smejda K, Stelmach I. Vitamin D supplementation in children may prevent asthma exacerbation triggered by acute respiratory infection. *Journal of allergy and clinical immunology.* 2011;127(5):1294-1296.

12. Manaseki-Holland S, Qader G, Isaq Masher M, et al. Effects of vitamin D supplementation to children diagnosed with pneumonia in Kabul: a randomised controlled trial. *Tropical medicine & international health.* 2010;15(10):1148-1155.

13. Martineau AR, Hanifa Y, Hooper RL, Witt KD, Patel M, Syed A. Increased risk of upper respiratory infection with addition of intermittent bolus-dose vitamin D supplementation to a daily low-dose regimen. *Thorax.* 2013;68(Suppl 3):A64 [s123]

14. McDonald CF, Zebaze RM, Seeman E. Calcitriol does not prevent bone loss in patients with asthma receiving corticosteroid therapy: a double-blind placebo-controlled trial. *Osteoporosis international.* 2006;17(10):1546-1551.

15. The Addition of Vitamin D to Fluticasone Propionate in the Management of Seasonal Allergic Rhinitis. *ClinicalTrialsgov [accessed 1 July 2013].* 2010.

16. Sazawal S, Dhingra U, Dhingra P, et al. Effects of fortified milk on morbidity in young children in north India: community based, randomised, double masked placebo controlled trial. *BMJ (Clinical research ed.).* 2007;334(7585):140.

17. Sneve M, Figenschau Y, Jorde R. Supplementation with cholecalciferol does not result in weight reduction in overweight and obese subjects. *European Journal of Endocrinology.* 2008;159(6):675-684.

18. Wu AC, Tantisira K, Li L, et al. Effect of vitamin D and inhaled corticosteroid treatment on lung function in children. *American Journal of Respiratory & Critical Care Medicine.* 2012;186(6):508-513.
