# Supplementary material for: Effect of Vitamin D3 Supplementation on Respiratory Tract Infections in Healthy Individuals: A Systematic Review and Meta-Analysis of Randomized Controlled Trials
Source: PLoS One. 2016 Sep 15;11(9):e0162996. doi: 10.1371/journal.pone.0162996 (PMC5025082; doi:10.1371/journal.pone.0162996)
Supplement: S1 Table — (DOCX) [file pone.0162996.s008.docx]

**Systematic review and meta-analysis on the effect of vitamin D supplementation on respiratory tract infection in healthy individuals.**

Danielle Vuichard Gysin, Dyda Dao, Christian Michael Gysin, Lyubov Lytvyn, Mark Loeb

**S1 Table. List of excluded RCTs.**

| **Study** | **Reason for exclusion** |
| --- | --- |
| **Avenell, 2007 [1]** | Outcomes of interest were not specifically addressed. |
| **Bartram, 2003 [2]** | Non-healthy population, other micronutrients and none of the outcomes of interest assessed. |
| **Behnamfar, 2011 [3]** | Comparator was vitamin E |
| **Bergmann, 2012 [4]** | Of the included participants, there were 80% with either a documented immunoglobulin deficiency or CVID and approximately half of the participants suffered from an underlying pulmonary disease including asthma, bronchiectasis or COPD |
| **Choudhary, 2012 [5]** | Population had underlying severe pneumonia. |
| **Fort, 2015 [6]** | Extremely preterm infants were deemed as non-healthy population. |
| **Gianni, 2014 [7]** | Non-healthy population (preterm infants) and low dose vitamin D in control group. |
| **Goldring, 2013 [8]** | Studied indirect effect: the pregnant mother and not the baby received the intervention |
| **Jain, 2002 [9]** | Comparator was other vitamins or micronutrients |
| **Jorde, 2012 [10]** | Non-healthy population. Authors state in their discussion section: "only a few of our subjects were completely healthy", ..."results may not apply for a more healthy population." |
| **Majak, 2011 [11]** | Non-healthy population. |
| **Manaseki, 2010 [12]** | Studied children admitted to hospital with pneumonia. |
| **Martineau, 2013 [13]** | Low-dose vitamin D as comparator. |
| **McDonald, 2006 [14]** | Non-healthy population. |
| **No author, trial no. NCT01103934 [15]** | Study results available (unpublished) but none of the outcomes of interest investigated. |
| **Sazawal, 2007 [16]** | other vitamins or micronutrients as comparator |
| **Sneve, 2008 [17]** | None of the outcomes of interest addressed. |
| **Wu, 2012 [18]** | Non-healthy population. |

**References of excluded randomized controlled trials:**

1. Avenell A, Cook JA, Maclennan GS, Macpherson GC. Vitamin D supplementation to prevent infections: a sub-study of a randomised placebo-controlled trial in older people (RECORD trial, ISRCTN 51647438). Age and ageing. 2007;36(5):574-7. doi: 10.1093/ageing/afm091. PubMed PMID: 17702768.

2. Bartram SA, Peaston RT, Rawlings DJ, Francis RM, Thompson NP. A randomized controlled trial of calcium with vitamin D, alone or in combination with intravenous pamidronate, for the treatment of low bone mineral density associated with Crohn's disease. Alimentary Pharmacology and Therapeutics. 2003;18(11-12):1121-7. PubMed PMID: 2004008707.

3. Behnamfar Z, Mehrdad S, Zahra B. Effect of maintenance dose (30000 unit per month) 25- Hydroxyvitamin D on Upper Respiratory Tract Infection in children of day care center. European Journal of Medical Research. 2011;16:49. PubMed PMID: 71294459.

4. Bergman P, Norlin AC, Hansen S, Rekha RS, Agerberth B, Bjorkhem-Bergman L, et al. Vitamin D3 supplementation in patients with frequent respiratory tract infections: A randomised and double-blind intervention study. BMJ open [Internet]. 2012; 2(6). Available from: <http://onlinelibrary.wiley.com/o/cochrane/clcentral/articles/175/CN-00912175/frame.html>

<http://bmjopen.bmj.com/content/2/6/e001663.full.pdf>.

5. Choudhary N, Gupta P. Vitamin D supplementation for severe pneumonia--a randomized controlled trial. Indian pediatrics [Internet]. 2012; 49(6):[449-54 pp.]. Available from: <http://onlinelibrary.wiley.com/o/cochrane/clcentral/articles/687/CN-00840687/frame.html>.

6. Fort P, Salas AA, Ambalavanan N. Randomized clinical trial of vitamin D supplementation in extremely preterm infants. Journal of Investigative Medicine. 2015;63 (2):417. PubMed PMID: 71769839.

7. Gianni ML, Roggero P, Amato O, Picciolini O, Piemontese P, Liotto N, et al. Randomized outcome trial of nutrient-enriched formula and neurodevelopment outcome in preterm infants. BMC Pediatrics. 2014;14(1). PubMed PMID: 2014287774.

8. Goldring ST, Griffiths CJ, Martineau AR, Robinson S, Yu C, Poulton S. Prenatal Vitamin D Supplementation and Child Respiratory Health: A Randomised Controlled Trial. PloS one [Internet]. 2013; 8(6):[e66627 p.]. Available from: <http://onlinelibrary.wiley.com/o/cochrane/clcentral/articles/208/CN-00866208/frame.html>

<http://www.ncbi.nlm.nih.gov/pmc/articles/PMC3691177/pdf/pone.0066627.pdf>.

9. Jain AL. Influence of vitamins and trace-elements on the incidence of respiratory infection in the elderly. Nutrition Research. 2002;22(1-2):85-7. PubMed PMID: 2002067526.

10. Jorde R, Witham M, Janssens W, Rolighed L, Borchhardt K, Boer IH, et al. Vitamin D supplementation did not prevent influenza-like illness as diagnosed retrospectively by questionnaires in subjects participating in randomized clinical trials. Scandinavian journal of infectious diseases [Internet]. 2012; 44(2):[126-32 pp.]. Available from: <http://onlinelibrary.wiley.com/o/cochrane/clcentral/articles/552/CN-00836552/frame.html>

<http://www.ncbi.nlm.nih.gov/pmc/articles/PMC3917712/pdf/nihms548537.pdf>.

11. Majak P, Olszowiec-Chlebna M, Smejda K, Stelmach I. Vitamin D supplementation in children may prevent asthma exacerbation triggered by acute respiratory infection. Journal of allergy and clinical immunology [Internet]. 2011; 127(5):[1294-6 pp.]. Available from: <http://onlinelibrary.wiley.com/o/cochrane/clcentral/articles/623/CN-00787623/frame.html>

<http://www.jacionline.org/article/S0091-6749(10)01957-3/pdf>.

12. Manaseki-Holland S, Qader G, Isaq Masher M, Bruce J, Zulf Mughal M, Chandramohan D, et al. Effects of vitamin D supplementation to children diagnosed with pneumonia in Kabul: a randomised controlled trial. Tropical medicine & international health [Internet]. 2010; 15(10):[1148-55 pp.]. Available from: <http://onlinelibrary.wiley.com/o/cochrane/clcentral/articles/287/CN-00760287/frame.html>

<http://onlinelibrary.wiley.com/store/10.1111/j.1365-3156.2010.02578.x/asset/j.1365-3156.2010.02578.x.pdf?v=1&t=i6377127&s=a0d5d6b21dfce0fd5ef05217828e68b2b4fc483a>.

13. Martineau AR, Hanifa Y, Hooper RL, Witt KD, Patel M, Syed A. Increased risk of upper respiratory infection with addition of intermittent bolus-dose vitamin D supplementation to a daily low-dose regimen [Abstract]. Thorax [Internet]. 2013; 68(Suppl 3):[A64 [s123]? p.]. Available from: <http://onlinelibrary.wiley.com/o/cochrane/clcentral/articles/610/CN-00990610/frame.html>

<http://thorax.bmj.com/content/68/Suppl_3/A64.1.full.pdf>.

14. McDonald CF, Zebaze RM, Seeman E. Calcitriol does not prevent bone loss in patients with asthma receiving corticosteroid therapy: a double-blind placebo-controlled trial. Osteoporosis international [Internet]. 2006; 17(10):[1546-51 pp.]. Available from: <http://onlinelibrary.wiley.com/o/cochrane/clcentral/articles/780/CN-00608780/frame.html>

<http://link.springer.com/article/10.1007%2Fs00198-006-0158-2>.

15. The Addition of Vitamin D to Fluticasone Propionate in the Management of Seasonal Allergic Rhinitis. ClinicalTrialsgov [accessed 1 July 2013] [Internet]. 2010. Available from: <http://onlinelibrary.wiley.com/o/cochrane/clcentral/articles/944/CN-00862944/frame.html>.

16. Sazawal S, Dhingra U, Dhingra P, Hiremath G, Kumar J, Sarkar A, et al. Effects of fortified milk on morbidity in young children in north India: community based, randomised, double masked placebo controlled trial. BMJ (Clinical research ed) [Internet]. 2007; 334(7585):[140 p.]. Available from: <http://onlinelibrary.wiley.com/o/cochrane/clcentral/articles/000/CN-00574000/frame.html>

<http://www.bmj.com/content/bmj/334/7585/140.full.pdf>.

17. Sneve M, Figenschau Y, Jorde R. Supplementation with cholecalciferol does not result in weight reduction in overweight and obese subjects. European Journal of Endocrinology. 2008;159(6):675-84. PubMed PMID: 2009052678.

18. Wu AC, Tantisira K, Li L, Fuhlbrigge AL, Weiss ST, Litonjua A, et al. Effect of vitamin D and inhaled corticosteroid treatment on lung function in children. American Journal of Respiratory & Critical Care Medicine. 2012;186(6):508-13. PubMed PMID: 22798322; PubMed Central PMCID: PMCPMC3480528.
