# Supplementary material for: Effect of Vitamin D3 Supplementation on Respiratory Tract Infections in Healthy Individuals: A Systematic Review and Meta-Analysis of Randomized Controlled Trials
Source: PLoS One. 2016 Sep 15;11(9):e0162996. doi: 10.1371/journal.pone.0162996 (PMC5025082; doi:10.1371/journal.pone.0162996)
Supplement: S2 Table — (DOCX) [file pone.0162996.s009.docx]

**Systematic review and meta-analysis on the effect of vitamin D supplementation on respiratory tract infection in healthy individuals.**

Danielle Vuichard Gysin, Dyda Dao, Christian Michael Gysin, Lyubov Lytvyn, Mark Loeb

**S2 Table. GRADE level of evidence and summary of findings of Vitamin D for the prevention of RTIs**

| **Quality assessment** | | | | | | | **Summary of Findings** | | | | |
| --- | --- | --- | --- | --- | --- | --- | --- | --- | --- | --- | --- |
| **Participants (studies) Follow up** | **Risk of bias** | **Inconsistency** | **Indirectness** | **Imprecision** | **Publication bias** | **Overall quality of evidence** | **Study event rates (%)** | | **Relative effect** (95% CI) | **Anticipated absolute effects** | |
|  |  |  |  |  |  |  | **With Control** | **With Vitamin D versus Control** |  | **Risk with Control** | **Risk difference with Vitamin D versus Control** (95% CI) |
| **Clinical Respiratory Tract Infection (RTI) (at least one episode):** | | | | | | | | | | | |
| 6985 (14 studies) 17 weeks | no serious risk of bias | serious^1^ | no serious indirectness | no serious imprecision | strongly suspected^2^ | ⊕⊕⊝⊝ **LOW**^1,2^ due to inconsistency, publication bias | 1988/3291  (60.4%) | 2089/3694  (56.6%) | **RR 0.94**  (0.88 to 1) | **Study population** | |
|  |  |  |  |  |  |  |  |  |  | **604 per 1000** | **36 fewer per 1000** (from 72 fewer to 0 more) |
|  |  |  |  |  |  |  |  |  |  | **Moderate** | |
|  |  |  |  |  |  |  |  |  |  | **524 per 1000** | **31 fewer per 1000** (from 63 fewer to 0 more) |
| **Laboratory confirmed RTI (at least one episode):** | | | | | | | | | | | |
| 1392 (4 studies) 12.5 weeks | no serious risk of bias | serious^1^ | no serious indirectness | no serious imprecision | strongly suspected^3^ | ⊕⊕⊝⊝ **LOW**^1,3^ due to inconsistency, publication bias | 216/660 (32.7%) | 211/732 (28.8%) | **RR 0.9** (0.68 to 1.21) | **Study population** | |
|  |  |  |  |  |  |  |  |  |  | **327 per 1000** | **33 fewer per 1000** (from 105 fewer to 69 more) |
|  |  |  |  |  |  |  |  |  |  | **Moderate** | |
|  |  |  |  |  |  |  |  |  |  | **271 per 1000** | **27 fewer per 1000** (from 87 fewer to 57 more) |
| **Mean duration (in days) of RTI (any episode):** (Better indicated by lower values) | | | | | | | | | | | |
| 1641 (6 studies) 14.5 weeks | no serious risk of bias | no serious inconsistency | no serious indirectness | serious^4^ | strongly suspected^3^ | ⊕⊕⊝⊝ **LOW**^3,4^ due to imprecision, publication bias | 820 | 821 | **-** | The mean duration of RTI in the intervention groups was **0.06 lower** (0.29 lower to 0.18 higher) | |
| **Mean number of sick days** (Better indicated by lower values) | | | | | | | | | | | |
| 972 (3 studies) 26 weeks | no serious risk of bias | serious^1^ | no serious indirectness | serious^4^ | strongly suspected^3^ | ⊕⊝⊝⊝ **VERY LOW**^1,3,4^ due to inconsistency, imprecision, publication bias | 465 | 507 | **-** |  | The mean number of sick days in the intervention groups was **0.06 higher** (0.41 lower to 0.54 higher) |
| **Severity of RTI** | | | | | | | | | | | |
| 585 (5 studies) 12 weeks | no serious risk of bias | no serious inconsistency | serious^5^ | no serious imprecision | reporting bias strongly suspected^3^ | ⊕⊕⊝⊝ **LOW**^3,4^ due to indirectness, publication bias | 0/297  (0%) | 0/288  (0%) | **OR 0.95**  (0.76 to 1.18) | **Study population** | |
|  |  |  |  |  |  |  |  |  |  | - | - |

^1^ Unexplained heterogeneity
^2^ Visual inspection of Funnel plot and Egger's test strongly suggest publication bias.
^3^ Low number of published studies did not allow formal assessment of publication bias.
^4^ Large confidence intervals.
^5^ Various definitions were applied to measure this outcome.
